# Investigating Interleukin-6 Levels in Type 2 Diabetes Mellitus Patients With and Without Diabetic Nephropathy

**DOI:** 10.7759/cureus.67014

**Published:** 2024-08-16

**Authors:** Vutukuru Kalyan Kumar Reddy, Govind Shiddapur, Nilesh Jagdale, Mohith Prakash Kondapalli, Saimounika Adapa

**Affiliations:** 1 Department of General Medicine, Dr. D. Y. Patil Medical College, Hospital and Research Centre, Dr. D. Y. Patil Vidyapeeth (Deemed to be University), Pune, IND

**Keywords:** proinflammatory cytokine, interleukin-6 (il-6), diabetic nephropathy (dn), type 2 diabetes mellitus (t2dm), urine albumin-to-creatinine ratio (uacr)

## Abstract

Background and objective: Diabetic nephropathy (DN), a severe complication affecting 40% of diabetic individuals, is a leading cause of chronic kidney disease (CKD). It involves a progressive increase in urinary albumin and a decline in the glomerular filtration rate. Early detection and intervention are crucial to preventing CKD progression. The current marker, albuminuria, measured as the urine albumin-to-creatinine ratio (UACR), has limitations, highlighting the need for alternative biomarkers. Researchers have linked the proinflammatory cytokine interleukin-6 (IL-6) to the progression of DN, observing elevated levels in DN patients compared to those without DN. IL-6 also regulates glucose metabolism, promoting insulin effectiveness and secretion. Inflammation and glucose control are two things that IL-6 does. This makes it a promising biomarker and therapeutic target for DN and type 2 diabetes mellitus (T2DM). This study focuses on IL-6 levels in T2DM patients with and without DN.

Methods and materials: From September 2022 to June 2024, the Department of General Medicine, Dr. D. Y. Patil Medical College, Hospital and Research Centre, Dr. D. Y. Patil Vidyapeeth (Deemed to be University), Pune, conducted an observational cross-sectional comparative study on 80 T2DM patients, with 40 in group A (cases = T2DM patients with DN) and 40 in group B (controls = T2DM patients without DN). The study included patients with T2DM between the ages of 40 and 80. The study excludes conditions such as diabetic ketoacidosis, patients with end-stage renal disease, and conditions that increase IL-6, such as COVID-19. The study excluded autoimmune conditions with elevated IL-6, such as rheumatoid arthritis, systemic lupus erythematous, ankylosing spondylitis, psoriasis, and Crohn's disease. We obtained ethical approval and written consent from participants.

Results: In the current study, 61 patients (76.2%) were 60 years old or younger, while 19 patients (23.8%) were older than 60 years. Among the participants, 38 were females (47.5%) and 42 were males (52.5%). The case group, which consisted of 40 T2DM patients with DN, had a mean glycated hemoglobin (HbA1c) of 7.1700 ± 0.71044. In contrast, the control group, comprising 40 T2DM patients without DN, had a mean HbA1c of 6.8650 ± 0.57179. This difference was statistically significant, with a p value of 0.038. Additionally, the mean UACR in the case group was 134.34 ± 95.56, significantly higher than the control group's mean UACR of 22.32 ± 9.90. This difference was highly significant, with a p value of 0.001. Furthermore, the case group exhibited elevated mean IL-6 levels of 15.48 ± 4.27 compared to the control group's 7.02 ± 2.46, which is also highly significant, reflected by a p value of 0.001.

Conclusion: As the concentration of IL-6 rises in diabetic patients with nephropathy, this study suggests that IL-6 may have an effect on the development of DN. This cytokine is necessary for both the initiation and progression of the condition. Using IL-6 as a supportive diagnostic test could help rule out other potential causes of DN in T2DM. Moreover, this marker does not require invasive procedures, and early measurement may help reduce mortality and morbidity.

## Introduction

Diabetic nephropathy (DN) is an illness marked by a steady rise in urine albumin and a decrease in glomerular filtration rate, making it a serious diabetes consequence. DN affects around 40% of people with diabetes, making it a major cause of chronic kidney disease (CKD) [1]. We routinely use albuminuria, evaluated as the urine albumin-to-creatinine ratio (UACR), as an early indicator of DN. However, studies have highlighted the limitations of albuminuria, including its lack of correlation with CKD stages in some patients [2]. This underscores the need for alternative biomarkers that can accurately detect DN at an early stage, thereby preventing the progression of CKD. Researchers have investigated various biomarkers such as glycated hemoglobin (HbA1c), interleukin (IL)-6, IL-18, tumor necrosis factor-alpha, and serum cystatin C, including those indicating tubular damage and inflammation [3].

Multiple studies and research have demonstrated that IL-6 signaling contributes to the progression of DN [4-7]. Type 2 diabetes mellitus (T2DM) patients with DN have been observed to have higher levels of IL-6 in the bloodstream than T2DM patients without DN [8]. This suggests a significant link between IL-6 and DN development and progression. Due to its various functions in regulating glucose balance [9], IL-6 is a compelling focus in diabetes research. IL-6 promotes insulin's effectiveness by enhancing glucose removal in the liver and skeletal muscle during exercise.

IL-6 is a versatile cytokine that plays a critical role in T2DM development. It achieves this by improving the body's response to insulin, regulating the body's glucose balance, and promoting insulin release. AMP-activated protein kinase activation leads to an increase in insulin-driven glucose elimination, resulting in elevated levels of IL-6, which are commonly associated with increased insulin resistance, a hallmark of T2DM. Administering IL-6 infusions in healthy individuals at a rate of 5 μg per hour has been demonstrated to enhance the ability of insulin to eliminate glucose efficiently [10].

In addition, the skeletal muscle releases IL-6 during exercise [11]. Moreover, research carried out on individuals has shown that IL-6 promotes the secretion of insulin by promoting the production of glucagon-like peptide-1 [12]. IL-6 is a proinflammatory cytokine that stimulates inflammation and contributes to the pathophysiology of T2DM. It impacts the regulation of glucose levels and balance in various regions of the body, such as neuroendocrine cells, adipocytes, peripheral tissues, and pancreatic islets [13]. Mounting evidence points to a significant association between elevated IL-6 levels and the onset of T2DM [14-16]. IL-6, along with other proinflammatory substances, has a crucial role in developing and advancing insulin resistance and T2DM. This finding highlights IL-6's significant role in inducing low-grade inflammation in specific tissues or throughout the body, thereby promoting the development of insulin resistance and T2DM [1,17].

IL-6 is responsible for facilitating inflammatory reactions, which substantially impact the advancement of DN. The detrimental effects of IL-6 in DN are facilitated by its classic signaling and trans-signaling mechanisms. The conventional method entails binding IL-6 to its receptor (IL-6R), located on specific cell types, such as hepatocytes and certain leukocytes. Subsequently, the receptor-ligand complex connects with glycoprotein 130. These pathways play a role in stimulating inflammatory responses, cellular growth, and survival. This mechanism, known as the classic signaling pathway, is limited to cells expressing membrane-bound IL-6R [18].

Trans-signaling, on the other hand, involves IL-6 interacting with a soluble form of IL-6R, allowing the cytokine to affect a broader range of cells that do not express the membrane-bound receptor. This mechanism is particularly relevant in the context of chronic inflammation, as it amplifies the inflammatory response by extending the range of IL-6 activity [19].

In DN, IL-6 trans-signaling contributes to the recruitment and activation of additional inflammatory cells in the kidney, exacerbating tissue damage and fibrosis. Mesangial cells, a type of kidney cell involved in the structure and function of the glomerulus, are particularly affected by IL-6. Elevated IL-6 levels lead to mesangial cell proliferation and the production of extracellular matrix components, contributing to mesangial expansion and glomerulosclerosis [20].

The study aims to investigate IL-6 levels in patients with T2DM, focusing on the distinction between those with and without DN. To achieve this, patients with T2DM are categorized into two groups: those with DN and those without. Within these groups, the investigators measure UACR, HbA1c, and IL-6 levels. Furthermore, the research seeks to assess the association among UACR, HbA1c, and IL-6 levels and the severity of DN, aiming to provide a deeper understanding of the inflammatory and metabolic markers involved in the progression of DN.

## Materials and methods

Study design and setting

The present study was an observational cross-sectional comparative study conducted at the Department of Medicine, Dr. D. Y. Patil Medical College, Hospital and Research Centre, Dr. D. Y. Patil Vidyapeeth (Deemed to be University), Pune. The study period extended from September 2022 to June 2024. Before the commencement of the investigation, approval was obtained from the Institute’s Scientific and Ethics Committee (ethical committee clearance number: IESC/PGS/2022/02). Written consent forms were provided to participants in their languages, ensuring they understood the study’s goals, procedures, and potential risks.

Inclusion and exclusion criteria

The study included patients with T2DM between the ages of 40 and 80. The study excludes conditions such as diabetic ketoacidosis, patients with end-stage renal disease, and conditions that increase IL-6, such as COVID-19. The study also excluded autoimmune conditions with elevated IL-6, such as rheumatoid arthritis, systemic lupus erythematous, ankylosing spondylitis, psoriasis, and Crohn's disease.

Sample size

Based on the study conducted by Huong et al. [21], this study examines the mean and standard deviation (SD) of IL-6 levels in both diabetic and nondiabetic patients. With an SD of 0.64 for group A (cases = patients with DN) and 0.57 for group B (controls = patients without DN), a difference of 0.4 at a significance level of 5%, and a power of 80%, we calculate the sample size to be 80, with 40 individuals in group A and 40 in group B. WinPepi version 11.65 (updated by J. H. Abramson) was the software used.

Data collection and consent

This study aimed to investigate the levels of IL-6 in patients diagnosed with T2DM, focusing on the comparison between those who had developed DN and those who had not. Participants were recruited, and their involvement included a thorough review of their medical history and a series of clinical and laboratory investigations. Each participant’s age, gender, height, weight, and body mass index were recorded. Detailed information regarding the duration of their T2DM and their family history of diabetes was collected. We evaluated DN using UACR. These measurements were critical in assessing kidney function and nephropathy severity. Blood samples were collected to measure fasting blood sugar, postprandial blood sugar, and HbA1c levels, which provided an overview of the patient’s glucose control. The primary focus of the blood investigation was the measurement of serum IL-6 levels, as this cytokine is a marker of inflammation and may play a role in the pathogenesis of DN.

Participants were provided detailed information about the study, including its purpose, procedures, potential risks, and benefits. They were informed that their participation was voluntary and that they could withdraw at any time without impacting their medical care. The confidentiality of their data was assured, and their identity was not disclosed in any publications resulting from the study. Each participant was required to sign a consent form indicating that they had read and understood the information provided, had the opportunity to ask questions, and agreed to participate in the study.

By gathering comprehensive data on medical history, clinical examination results, and relevant blood investigations, this study aimed to enhance our understanding of the role of IL-6 in T2DM and its complications, potentially informing better management strategies for patients with DN.

Statistical analysis

A spreadsheet (Microsoft Excel 2010) was used to input all of the collected information, which was subsequently uploaded to the data editor in SPSS 20 (SPSS Inc., Chicago, IL). The descriptive statistics included the computation of percentages, averages, and SDs. The study employed a chi-square test and a student t-test to determine statistical significance. To be more precise, we set a 95% CI and a 5% p value.

## Results

Age distribution

Of the total number of patients, 61 (76.2%) are aged 60 or below, while 19 (23.8%) are above 60. Within the case group, the majority of patients, 33 (82.5%), were 60 years old or younger, while seven patients (17.5%) were older than 60. In the control group, 28 patients (70%) were 60 years old or younger, and 12 (30%) were older than 60. According to the chi-square test, age and group categorization did not significantly correlate, which produced a p value of 0.189 (Table 1).

**Table 1:** Age distribution in both the groups n: number

Age (years)	Case (group A), n (%)	Control (group B), n (%)	Total, n (%)	p value
≤60	33 (82.5%)	28 (70.0%)	61 (76.2%)	0.189
>60	7 (17.5%)	12 (30.0%)	19 (23.8%)	
Total	40 (100.0%)	40 (100.0%)	80 (100.0%)	

Gender distribution

Of the total patients, 38 (47.5%) were females and 42 (52.5%) were males. In this case group, 22 (55%) patients were female, whereas 18 (45%) were male. The control group comprised 16 females (40%) and 24 males (60%). The gender parameter did not exhibit any statistically significant correlation with the study's case or control groups, as indicated by the chi-square test result of a p value of 0.179 (Table 2).

**Table 2:** Gender distribution in both the groups n: number

Gender	Case (group A), n (%)	Control (group B), n (%)	Total, n (%)	p value
Female	22 (55.0%)	16 (40.0%)	38 (47.5%)	0.179
Male	18 (45.0%)	24 (60.0%)	42 (52.5%)	
Total	40 (100.0%)	40 (100.0%)	80 (100.0%)	

Mean value of HbA1c

The case group's mean HbA1c was 7.1700 ± 0.71044, while the control group's was 6.8650 ± 0.57179. The Student's t-test yielded a p value of 0.038, indicating a statistically significant difference in the HbA1c values between the case and control groups (Table 3).

**Table 3:** Mean value of HbA1c in both the groups HbA1c: glycated HbA1c; n: number

Parameters	Groups	n	Mean	Standard deviation	p value
HbA1c	Case (group A)	40	7.1700	0.71044	0.038
	Control (group B)	40	6.8650	0.57179	

Mean value of UACR

The case group's mean UACR was 134.34 ± 95.56, while the control group's was 22.32±9.90. The student's t-test p value of 0.001 indicated a statistically significant difference between the UACRs in the case and control groups (Table 4).

**Table 4:** Mean value of UACR in both the groups UACR: urine albumin-to-creatinine ratio; n: number

Parameters	Groups	n	Mean	Standard deviation	p value
UACR	Case (group A)	40	134.3400	95.56467	0.001
	Control (group B)	40	22.3200	9.89774	

Mean value of IL-6 in both groups

In the case group, mean IL-6 levels were 15.48 ± 4.27, while in the control group, they were 7.02 ± 2.46. Using the student t-test, the association between the IL-6 levels in both the control and the case groups was statistically significant, with a p value of 0.001 (Table 5).

**Table 5:** Mean value of IL-6 in both the groups IL-6: interleukin-6; n: number

Parameters	Groups	n	Mean	Standard deviation	p value
IL-6	Case (group A)	40	15.4850	4.26984	0.001
	Control (group B)	40	7.0200	2.46527	

## Discussion

Among the participants of the present study, 61 patients (76.2%) were aged 60 years or below, whereas 19 patients (23.8%) were above the age of 60. Within the case group consisting of patients with DN, 33 patients (82.5%) were aged 60 or below, whereas seven patients (17.5%) were above 60. In contrast, the control group, which included patients without DN, had 28 patients (70%) who were 60 years old or younger and 12 patients (30%) older than 60. There was no statistically significant difference in the age distribution between the case and control groups, according to the chi-square test (p = 0.189). According to Huong et al. [21], a p value of 0.493 shows that there was no discernible difference in the age distribution between the case group and the control group. Both studies emphasize that age does not significantly distinguish between patients with DN and those without.

In the current study, the gender distribution among participants included 38 females (47.5%) and 42 males (52.5%). Specifically, the case group, which included patients with DN, comprised 18 males (45%) and 22 females (55%). In contrast, the control group, consisting of patients without DN, had 24 males (60%) and 16 females (40%). A p value of 0.179 indicated that there was no statistically significant association between gender and the existence of DN, as determined by the statistical study. The research by Huong et al. [21], which investigated the role of serum IL-6 levels in DN, is consistent with this finding. Their study reported a p value of 0.678 when comparing the gender distribution between male and female T2DM patients with and without DN. Both studies demonstrate that gender does not significantly influence the development of DN.

The current study found that the case group, consisting of patients with DN, had a mean HbA1c level of 7.1700 ± 0.71044. The observed value was greater than the control group, with a lower average HbA1c concentration. The statistical study demonstrated a noteworthy association between HbA1c levels and the presence of DN, with a p value of 0.038. Furthermore, a study conducted by Huong et al. [21] found a p value of 0.024 when comparing HbA1c levels between individuals with T2DM who had DN and those who did not have DN. This p value suggests a substantial difference between the two groups. In their study, patients without DN had an average HbA1c level of 6.75% with an SD of 1.40, whereas patients with DN had an average HbA1c level of 7.20% with an SD of 1.80. The p values in both studies, 0.038 in the current study and 0.024 in the study by Huong et al. [21], indicate statistically significant differences in HbA1c levels between the case and control groups. These findings collectively highlight that individuals with T2DM and DN have notably elevated HbA1c levels, underscoring the importance of long-term blood glucose regulation. These findings emphasize the importance of maintaining optimal HbA1c levels to prevent DN development and progression.

In this current study, the mean UACR for the case group was 134.34 ± 95.56, whereas for the control group, it was 22.32 ± 9.90. It shows that UACR levels are significantly higher in people with DN compared to those who do not have DN (p= 0.001). This shows that UACR could be used as a biomarker to find and track DN early on. Similarly, Ying et al. [22] investigated the predictive value of UACR in CKD patients. They concluded that it strongly predicts adverse renal outcomes such as end-stage kidney disease (ESKD) and significant declines in estimated glomerular filtration rate, with p values less than 0.001 indicating high statistical significance. Together, these studies emphasize UACR's utility in general CKD prognosis and specific DN management, supported by robust statistical evidence.

Our study observed that the case group had a mean IL-6 level of 15.48 ± 4.27 pg/mL, significantly higher than the control group's mean IL-6 level of 7.02 ± 2.46 pg/mL, with a statistically significant correlation (p = 0.001). Similarly, Sanchez-Alamo et al. [23] found that elevated serum IL-6 levels are significantly associated with the progression of kidney disease in DN patients. Their study underscores that higher IL-6 levels are linked to a greater risk of advancing to ESKD, reinforcing IL-6 as a critical biomarker for DN.

Comparatively, Huong et al. [21] reported mean IL-6 levels of 18.80 ± 22.96 pg/mL in DN patients and 10.03 ± 10.44 pg/mL in patients without DN, with a significant p value of 0.013. The larger SD in DN patients (22.96 pg/mL) versus those without DN (10.44 pg/mL) suggests considerable variability in IL-6 levels, likely due to different stages of DN and individual inflammatory responses. This variability aligns with our findings, where the case group's IL-6 levels were more variable than those of the control group, indicating that IL-6 reflects the underlying heterogeneity in disease severity.

Our study and that of Sanchez-Alamo et al. [23] highlight the potential of IL-6 as a noninvasive biomarker for detecting and monitoring DN progression. Elevated IL-6 levels not only indicate increased inflammatory activity but also correlate with worse renal outcomes in diabetic patients. The significant differences in IL-6 levels between groups underscore its role in the inflammatory processes associated with diabetes complications, suggesting that monitoring IL-6 levels could provide valuable insights into disease progression and guide therapeutic interventions.

Limitations

The small sample size was a limitation of this study. It is worth doing a comprehensive prospective cohort study at large scales to reveal the diagnostic value of IL-6 in type 2 DN. A single medical center conducted this research, potentially limiting the generalizability of the findings to other populations and settings. We need multicenter studies involving diverse demographic groups to validate the results. Excluding patients with conditions that elevate IL-6 levels, such as autoimmune diseases and COVID-19, may introduce selection bias. This exclusion criterion could limit the applicability of the findings to the broader diabetic population, some of whom may have comorbid conditions affecting IL-6 levels.

## Conclusions

As the concentration of IL-6 rises in diabetic patients with nephropathy, this study suggests that IL-6 plays a significant role in the development and progression of DN. IL-6 is crucial for initiating and advancing DN, making it a valuable biomarker. Administering IL-6 as a supportive diagnostic test can help rule out other potential causes of DN in patients with T2DM. Utilizing IL-6 as a marker offers the advantage of a noninvasive diagnostic approach, simplifying the early detection process. Early measurement and monitoring of IL-6 levels can provide critical insights into disease progression, allowing for timely intervention. By identifying and addressing DN at an early stage, healthcare providers can implement strategies to reduce associated mortality and morbidity. The study emphasizes IL-6's potential not only as a diagnostic tool but also as a target for therapeutic intervention in managing DN and improving patient outcomes.

## References

[1] Umanath K, Lewis JB (2018). Update on diabetic nephropathy: core curriculum 2018. Am J Kidney Dis.

[2] Tuttle KR, Bakris GL, Bilous RW (2014). Diabetic kidney disease: a report from an ADA Consensus Conference. Diabetes Care.

[3] Al-Rubeaan K, Siddiqui K, Al-Ghonaim MA, Youssef AM, Al-Sharqawi AH, AlNaqeb D (2017). Assessment of the diagnostic value of different biomarkers in relation to various stages of diabetic nephropathy in type 2 diabetic patients. Sci Rep.

[4] Navarro-González JF, Mora-Fernández C, Muros de Fuentes M, García-Pérez J (2011). Inflammatory molecules and pathways in the pathogenesis of diabetic nephropathy. Nat Rev Nephrol.

[5] Stenvinkel P, Ketteler M, Johnson RJ (2005). IL-10, IL-6, and TNF-alpha: central factors in the altered cytokine network of uremia--the good, the bad, and the ugly. Kidney Int.

[6] Ng DP, Nurbaya S, Ye SH, Krolewski AS (2008). An IL-6 haplotype on human chromosome 7p21 confers risk for impaired renal function in type 2 diabetic patients. Kidney Int.

[7] Papaoikonomou S, Tousoulis D, Tentolouris N, Miliou A, Papageorgiou N, Synetos A, Stefanadis C (2014). Assessment of the effects of 174G/C polymorphism on interleukin 6 genes on macrovascular complications in patients with type 2 diabetes mellitus. Int J Cardiol.

[8] Taslipinar A, Yaman H, Yilmaz MI (2011). The relationship between inflammation, endothelial dysfunction and proteinuria in patients with diabetic nephropathy. Scand J Clin Lab Invest.

[9] Tummala KS, Brandt M, Teijeiro A, Graña O, Schwabe RF, Perna C, Djouder N (2017). Hepatocellular carcinomas originate predominantly from hepatocytes and benign lesions from hepatic progenitor cells. Cell Rep.

[10] Carey AL, Steinberg GR, Macaulay SL (2006). Interleukin-6 increases insulin-stimulated glucose disposal in humans and glucose uptake and fatty acid oxidation in vitro via AMP-activated protein kinase. Diabetes.

[11] Pedersen BK, Febbraio MA (2008). Muscle as an endocrine organ: focus on muscle-derived interleukin-6. Physiol Rev.

[12] Ellingsgaard H, Hauselmann I, Schuler B (2011). Interleukin-6 enhances insulin secretion by increasing glucagon-like peptide-1 secretion from L cells and alpha cells. Nat Med.

[13] Hu FB, Meigs JB, Li TY, Rifai N, Manson JE (2004). Inflammatory markers and risk of developing type 2 diabetes in women. Diabetes.

[14] Akbari M, Hassan-Zadeh V (2018). IL-6 signalling pathways and the development of type 2 diabetes. Inflammopharmacology.

[15] Kristiansen OP, Mandrup-Poulsen T (2005). Interleukin-6 and diabetes: the good, the bad, or the indifferent?. Diabetes.

[16] Popko K, Gorska E, Stelmaszczyk-Emmel A (2010). Proinflammatory cytokines Il-6 and TNF-α and the development of inflammation in obese subjects. Eur J Med Res.

[17] Spranger J, Kroke A, Möhlig M (2003). Inflammatory cytokines and the risk to develop type 2 diabetes: results of the prospective population-based European Prospective Investigation into Cancer and Nutrition (EPIC)-Potsdam Study. Diabetes.

[18] Su H, Lei CT, Zhang C (2017). Interleukin-6 signaling pathway and its role in kidney disease: an update. Front Immunol.

[19] Donate-Correa J, Ferri CM, Sánchez-Quintana F (2020). Inflammatory cytokines in diabetic kidney disease: pathophysiologic and therapeutic implications. Front Med (Lausanne).

[20] Suzuki D, Miyazaki M, Naka R (1995). In situ hybridization of interleukin 6 in diabetic nephropathy. Diabetes.

[21] Huong PT, Nguyen CTT, Binh NG (2021). The role of serum interleukin-6 level in type 2 diabetic nephropathy. Res J Biotech.

[22] Ying T, Clayton P, Naresh C, Chadban S (2018). Predictive value of spot versus 24-hour measures of proteinuria for death, end-stage kidney disease or chronic kidney disease progression. BMC Nephrol.

[23] Sanchez-Alamo B, Shabaka A, Cachofeiro V, Cases-Corona C, Fernandez-Juarez G (2022). Serum interleukin-6 levels predict kidney disease progression in diabetic nephropathy. Clin Nephrol.

